# LncRNA DARS-AS1 aggravates the growth and metastasis of hepatocellular carcinoma via regulating the miR-3200-5p-Cytoskeleton associated protein 2 (CKAP2) axis

**DOI:** 10.1080/21655979.2021.1982272

**Published:** 2021-10-27

**Authors:** Yanqing Feng, Gang Wei, Linfei Zhang, Huadong Zhou, Wei Wang, Peng Guo, Caitao Cheng, Lei Ji, Qinghe Cai, Yong Feng, Huahua Tu

**Affiliations:** a Department of Hepatobiliary Surgery, Renmin Hospital, Hubei University of Medicine, Shiyan, Hubei, China; b Department of Gastroentrology, Renmin Hospital, Hubei University of Medicine, Shiyan, Hubei, China

**Keywords:** Hepatocellular carcinoma, DARS-AS1, CKAP2, FAK-ERK pathway, miR-3200-5p, metastasis

## Abstract

Accumulating signs have found that long noncoding RNAs (lncRNAs) contribute to hepatocellular carcinoma (HCC). Here, we probed the effect and mechanism of lncRNA DARS-AS1 in HCC. The profiles of DARS-AS1 and Cytoskeleton associated protein 2 (CKAP2) in 50 HCC tissues and non-tumor tissues were examined by real-time quantitative polymerase chain reaction (RT-qPCR). DARS-AS1 and CKAP2 overexpression and/or knockdown cell models were established. The proliferation, apoptosis, invasion and epithelial-mesenchymal transition (EMT) were determined. CKAP2, and focal adhesion kinase (FAK)-extracellular signal-regulated kinase (ERK) was tested by Western blot (WB). The relationship between DARS-AS1 and CKAP2 was predicted by Bioinformatics, and the dual-luciferase reporter assay was applied to verify the targeting association between miR-3200-5p and DARS-AS1 and CKAP2. DARS-AS1 was overexpressed in HCC tissues (vs. that in non-tumor tissues) and was closely correlated with the patients’ tumor stage. DARS-AS1 facilitated HCC cell proliferation and hampered apoptosis. HCC cell migration and EMT were enhanced by DARS-AS1. DARS-AS1 up-regulated CKAP2, which aggravated HCC. Further investigation illustrated that either DARS-AS1 or CKAP2 activated FAK-ERK pathway, and miR-3200-5p was competitively restrained by DARS-AS1. miR-3200-5p exerted tumor-suppressive effects in HCC and inactivated CKAP2 and FAK-ERK pathway. All in all, this study corroborates that DARS-AS1 facilitates HCC proliferation and metastasis by regulating miR-3200-5p-mediated CKAP2, which provides a potential target for HCC diagnosis and treatment.

**Abbreviations:** CCK-8: cell counting kit-8; CKAP2: Cytoskeleton associated protein 2; cDNA:complementary DNA; DAPI: 4ʹ,6-diamidino-2-phenylindole; DARS-AS1: DARS1 antisense RNA 1; DEPC: diethyl pyrocarbonate; DMEM-F12: Dulbecco’s minimal essential medium/Ham’s-F12; EMT: epithelial-mesenchymal transition; ERK: extracellular signal-regulated kinase; FAK: focal adhesion kinase; FBS: fetal bovine serum; GAPDH: glyceraldehyde-3-phosphate dehydrogenase; HCC: hepatocellular carcinoma; HE: hematoxylin-eosin; IHC: Immunohistochemistry; LIHC: Liver hepatocellular carcinoma; lncRNAs: long noncoding RNAs; MIAT: lncRNA myocardial infarction-related transcripts; MT: Mutant; NC: negative control; PBS: phosphate-buffered saline; PMSF: Phenylmethylsulfonyl fluoride; PVDF: polyvinylidene difluoride; RT: room temperature; RT-qPCR: real-time quantitative polymerase chain reaction; SDS-PAGE: sodium dodecyl sulfate-polyacrylamide gel electrophoresis; SPF: specific pathogen-free; TMAP: tumor-associated microtubule-associated protein; TUNEL: TdT-mediated dUTP nick end labeling; V: volume; WT: wild type.

## 1. Introduction

Hepatocellular carcinoma (HCC) is a typical malignancy worldwide and the third-leading contributor to cancer-associated deaths, with high malignancy, early metastasis, fast infiltration growth, and unfavorable prognosis [1]. Most HCC patients are diagnosed at an advanced stage of cancer, with only 10–20% of patients being diagnosed at an early stage. To make matters worse, the curative effect of microwave ablation, radiofrequency ablation, and hepatic artery embolization are limited [2,3,4]. Targeted molecular-based therapies have become indispensable for treating HCC, and it is imperative to explore the deeper molecular mechanisms of HCC pathogenesis with a view to bringing new light to personalized treatment of HCC.

Long noncoding RNAs (lncRNAs) are longer than 200 nt. Despite their limited ability to encode proteins, lncRNAs contribute to various cellular biological processes [5,6]. In the past few years, emerging studies have stated that lncRNAs exert particular leverage in HCC occurrence and development. For instance, lncRNA myocardial infarction-related transcripts (MIAT) accelerates HCC cell proliferation and invasion [7]. Furthermore, lncRNA HANR facilitates HCC tumorigenesis and chemoresistance to adriamycin [8]. Similarly, as a lncRNA, DARS1 antisense RNA 1 (DARS-AS1)’s carcinogenesis has been testified in ovarian cancer, clear cell renal cell carcinoma, non-small cell lung cancer, and cervical cancer [9,10,11,12]. However, its carcinogenesis in HCC has rarely drawn public’s attention.

Cytoskeleton-associated protein 2 (CKAP2), also known as tumor-associated microtubule-associated protein (TMAP), is a novel microtubule-associated protein encoded by a gene situated in chromosome 13. CKAP2 is localized on microtubule-organizing centers and microtubules, where it has an essential role in cell mitosis. In this way, CKAP2 stabilizes microtubules and contributes to regulating cell division [13]. In particular, CKAP2 is up-regulated in glioma [14], osteosarcoma [15], breast cancer [16], and cervical cancer [17] and engaged in their progression. Interestingly, CKAP2 is also a potential predictor of widespread short-term recurrence of HCC [18]. Nevertheless, how CKAP2 affects HCC progression awaits further investigation.

In this study, we aimed to explore the regulatory effect and mechanism of DARS-AS1 on HCC cells. Our study substantiated that overexpression of DARS-AS1 amplified the malignant phenotypes of HCC cells, up-regulated CKAP2 and activated the focal adhesion kinase (FAK)-extracellular signal-regulated kinase (ERK) pathway. Besides, the bioinformatics analysis demonstrated that miR-3200-5p was a potential target of DARS-AS1 and CKAP2. Therefore, we speculate that DARS-AS1 regulates CKAP2 and the FAK-ERK pathway by targeting miR-3200-5p and exerts a crucial role in modulating HCC evolvement. We hope this study provides more knowledge for the diagnosis and treatment of HCC in its early stage.

## 2. Materials and methods

### 2.1 Clinical sample collection

Clinical data and tissue samples were obtained from HCC patients who received surgical treatment in Renmin Hospital of Hubei University of Medicine from February 2015 to July 2016. Resected HCC tissues and non-tumor tissues (at least 3 cm away from the lesion) were immediately stored in a refrigerator at −80°C [19]. The diagnosis and staging of HCC were confirmed by two senior pathologists in the Department of Pathology. The Ethics Committee of Renmin Hospital of Hubei University of Medicine authorized this study (approval number: XYRH2018024). All enrolled in the study, patients or their families, had confirmed their participation, with an informed consent signed.

### 2.2 Cell culture

Both human normal liver epithelial cell lines (THLE-3) and HCC cell lines (Huh-7, HCCLM3, HLE, MHCC97, and HCCLM6) were bought from the Cell Center of the Chinese Academy of Sciences (Shanghai, China). The DMEM-F12 medium (Thermo Fisher Hyclone, Utah, USA) comprising 10% fetal bovine serum (FBS, Hyclone, Logan, UT, USA) and 1% penicillin/streptomycin was applied to culture the cells under specific pathogen-free (SPF) conditions (37°C, 5% CO_2_, saturated humidity) [20]. The logarithmic growth cells were applied for subsequent experiments. The cells were treated with 0.25% trypsin (Thermo Fisher Hyclone, Utah, USA) and sub-cultured.

### 2.3 Cell transfection

Cell transfection was performed according to a previous study [21]. Huh-7 and HCC-LM3 cells in the logarithmic growth phase were adjusted to reach a fusion rate of 60%~70%. They were then inoculated in 12-well plates (2 × 10^5^ cells/well) and cultured with DMEM-F12 containing 10% FBS without antibiotics. The primary medium was discarded after cell adherence. DARS-AS1 and CKAP2 overexpression/knockdown models were set up in Huh-7/HCCLM3 cells, respectively. pcDNA3.1-DARS-AS1, pcDNA3.1-CKAP2 and its negative vector plasmid, sh-CKAP2 and its shRNA negative control (sh-NC) were added to each well. The sequences were shCKAP2#1: CCGGCCAATATGACTGCCACTACTACTCGAGTAGTAGTGGCAGTCATATTGGTTTTTTG; shCKAP2#2: CCGGCCCTGTTCACTTTACTAAATACTCGAGTATTTAGTAAAGTGAACAGGGTTTTTG; shNC:CCGGCCTAAGGTTAAGTCGCCCTCGCTCGAGCGAGGGCGACTTAACCTTAGGTTTTTG. Cell transfection was performed using lipofectamine 2000 (Thermo FisherScience, Waltham, MA, USA) as per the manufacturer’s instructions. Twenty-four hours after the transfection, the transfection reagent was discarded, and the complete medium was supplemented. After further culture for 24 hours, the expression of the corresponding molecules was determined by real-time quantitative polymerase chain reaction (RT-qPCR) or Western blot (WB) to verify the transfection efficiency.

### 2.4 Cell counting kit-8 (CCK-8) assay

We prepared Huh-7 and HCCLM3 cells (logarithmic growth phase) and got them inoculated into 96-well plates. After 24 hours of incubation, 10 μL CCK-8 reagent (MedChem Express, New Jersey, USA) was added to each well. Thereafter, the plates were kept in an incubator for four hours, and the absorbance at 450 nm was gauged by a spectrophotometer (Thermo Fisher Scientific GENESYS 10S UV-Vis) [22]. Similarly, HCC cell proliferation at other time points (48 and 72 hours) was determined after the transfection. All tests were done three times.

### 2.5 Colony formation assay

A colony formation assay was carried out for evaluating HCC cell proliferation [22]. With Huh-7 and HCCLM3 cells (logarithmic growth phase) available, we seeded them into a 60 mm dish containing the culture medium (500 cells per dish), incubated them after a mix-up, and replaced the medium every 3 to 4 days. After 12 days, the cells were washed with phosphate-buffered saline (PBS), secured with 4% paraformaldehyde, stained with crystal violet, and photographed. Ultimately, we adopted the Image-Pro Plus software (National Institutes of Health) to count colonies in each dish. All experiments were repeated in triplicate.

### 2.6 Transwell assay

Twenty-four hours after the transfection, cells were trypsinized, harvested, and resuspended with the serum-free DMEM-F12 complete medium (1 × 10^5^/mL). Cells were seeded into the upper chamber of a Transwell compartment (8 μm pore diameter), whose lower chamber was DMEM-F12 complete medium containing 500 μL 10% PBS. After incubating for six hours, the non-migrated cells on the membranes were wiped off, and the cells that migrated and adhered to the lower chamber were immobilized with 4% paraformaldehyde and stained with crystal violet [23]. Five representative high magnification fields on the membranes were randomly chosen to calculate the transmembrane cell number. The mean value of the three repetitive wells was used to indicate the migrative ability of tumor cells. All tests were conducted three times.

### 2.7 Cell immunofluorescence

Huh-7 and HCCLM3 cells overexpressing DARS-AS1 were collected and seeded into 24-well plates containing the culture medium (1 × 10^5^ cells/well). After 24 hours of culture, the cells were fixed with 4% paraformaldehyde (Beyotime, Wuhan, China) at room temperature (RT) for 15 min and then rinsed with PBS three times (5 min each time). Afterward, 0.2 mL/L Triton X-100 was added (4°C, 15 min). Then, cells were washed with PBS for 5 min × 3 times, and the TUNEL reaction solution (Abcam, ab66110) or the anti-CKAP2 antibody (Abcam, ab198188; 1:100) was added for incubation in the dark at 37°C for one hour. Cells were then cleared with PBS for 5 min × 3 times and incubated with the 4ʹ,6-diamidino-2-phenylindole (DAPI) dye (Beyotime, Wuhan, China) for 10 min at 37°C [24]. Finally, the cells were rinsed with PBS for 5 min × 3 times, mounted with glycerin, and observed under a fluorescence microscope.

### 2.8 RT-qPCR

The TRIzol reagent (Invitrogen, Carlsbad, CA, USA) was adopted to extract total RNAs in HCC tissues and cells [25]. The concentration and purity of the total RNA were measured by ultraviolet spectrophotometry. The complementary DNA (cDNA) synthesis was implemented with the RevertAid First Strand cDNA Synthesis Kit (Thermo Fisher Scientific, Waltham, MA, USA). RT-qPCR primers were designed according to GenBank, and glyceraldehyde-3-phosphate dehydrogenase (GAPDH) served as the endogenous control. DARS-AS1, forward, 5ʹ-CATCGGGACACGGAACTGG-3ʹ, reverse 5ʹ-TGCAAAGAACTGCAGAAGACAC-3ʹ, CKAP2, forward, 5ʹ-GCAAGATGCTAACATGCCCAA-3ʹ, reverse, 5ʹ-TGGCTTTAGGTATAGTGGCTGA-3ʹ. GAPDH forward: 5ʹ-TGCACCACCAACTGCTTAGC-3ʹ, reverse 5ʹ-GGCATGGACTGTGGTCATGAG-3ʹ, U6 forward: 5ʹ- TCATCAGAAACAGTGGAGGT-3ʹ, reverse 5ʹ- CATCCTTACACAGGAGCCAT-3ʹ, miR-3200-5p: forward 5ʹ-AAUCUGAGAAGGCGCACAAGGU-3ʹ, reverse 5ʹ- TGGTGTCGTGGAGTCG-3ʹ. The PCR system was 25 μL, including 2 μL cDNA, 12.5 μL 2 × SYBR Green (MedChemExpress, NJ, USA), 0.5 μL of 25 μmol/L forward and reverse primers and 9.5 μL diethyl pyrocarbonate (DEPC) solution. The PCR condition was pre-denaturation at 95°C for 5 min, 95°C for 15 s, 60°C for 30 s, and 72°C for 30 s, with a total of 40 cycles. All tests were repeated three times.

### 2.9 WB

The tissues or cells were collected and lyzed by RIPA (Beyotime Biotechnology, Shanghai, China) containing 1% Phenylmethylsulfonyl fluoride (PMSF) on ice, and the total protein was extracted. Then the protein was quantified by the BCA protein concentration kit (Beyotime Biotechnology, Shanghai, China) and isolated by sodium dodecyl sulfate-polyacrylamide gel electrophoresis (SDS-PAGE). After electrophoresis, the protein was transferred to polyvinylidene difluoride (PVDF) membranes under 200 mA constant current for 90 min. Subsequently, the membranes were blocked with 10% skimmed milk solution for two hours and incubated with diluted antibodies (Abcam; 1:1000) of CKAP2 (ab227214), p-FAK (ab4792), FAK (ab40794), p-ERK (ab50011), ERK (ab17942), E-cadherin (ab40772), N-cadherin (ab76011), Vimentin (ab92547), Caspase3 (ab227214), Bcl2 (ab182858), and Bax (ab92547) overnight at 4°C. The membranes were washed with TBST three times (15 min each time). After being rinsed with TBST three times (15 min/time), the membranes were subjected to incubation with the secondary antibody at RT for two hours, followed by rewashing with TBST three times. The protein was imaged with ECL chemiluminescence solution [26]. GAPDH was the internal reference. All tests were repeated three times.

### 2.10 Bioinformatics analysis

The binding relationships between miR-3200-5p and lncRNA DARS-AS1 and CKAP2 were predicted by the online database Starbase database (http://starbase.sysu.edu.cn/). Venny 2.1 (https://bioinfogp.cnb.csic.es/tools/venny/index.html) was employed for analyzing the common targets of DARS-AS1 and CKAP2. GEPIA database (http://gepia.cancer-pku.cn/) was applied for probing the expression correlation between DARS-AS1 CKAP2, and CKAP2 expression in LIHC (Liver hepatocellular carcinoma). GEPIA database is a newly developed interactive web server for analyzing the RNA sequencing expression data of 9,736 tumors and 8,587 non-tumor samples from the TCGA and the GTEx projects, using a standard processing pipeline [27]. The median expression of CKAP2 serves as the cutoff value in survival analysis.

### 2.11 Dual-luciferase reporter assay

We designed and synthesized wild-type (WT) and mutant (MT) luciferase reporter vectors (DARS-AS1-WT, DARS-AS1-MT, CKAP2-WT, and CKAP2-MT) (Promega, Madison, WI, USA) based on the binding sites of DARS-AS1 and CKAP2 with miR-3200-5p predicted by the Starbase (http://starbase.sysu.edu.cn/). Huh7 cells (5 × 10^4^) were inoculated in 48-well plates and incubated at 37°C for 24 hours. Then they were co-transfected with DARS-AS1-WT, DARS-AS1-MT, CKAP2-WT, CKAP2-MT and miR-3200-5p mimics or negative control using lipofectamine 2000. Forty-eight hours later, the luciferase activity was tested by a dual-luciferase reporter gene assay system (Promega) [24]. All experiments were made in triplicate.

### 2.12 Tumor formation experiment in nude mice

Twenty BALB/cASlacnt mice (male, 6–8 weeks old) were bought from the Experimental Animal Center of Wuhan University. All the mice were reared under SPF conditions and had free access to water and food. Huh7 cells transfected with DARS-AS1 overexpression plasmids or negative vectors were trypsinized to make single-cell suspensions (2× l0^7^/mL). Each nude mouse was injected with 0.1 mL single-cell suspension under the left and right armpits and then reared under SPF condition [24]. The mice’s diet, activity and systemic condition were observed. From day 7, the long diameter (a) and short diameter (b) of the tumor were determined weekly, and the volume (V) = a× b^2^/2. Finally, the mice were executed, and the tumors were weighed. The mice’s lung tissues were collected, and tumor metastasis was observed by hematoxylin-eosin (HE) staining. This study was approved by the Ethics Committee of Renmin Hospital of Hubei University of Medicine (approve number: XYRH2018025). All experimental procedures were done to minimize damage to the mice.

### 2.13 Immunohistochemistry (IHC)

The exfoliated tumor tissues were immobilized with 4% paraformaldehyde for four hours, paraffin-embedded, and sectioned (4 μM thick). The sections were then subjected to dewaxing, antigenic repair, and blocking in 5% FBS. Afterward, the antibodies of E-cadherin (Abcam, ab40772, 1:500), Ki67 (Abcam, ab15580, 1:500), Vimentin (Abcam, ab92547, 1:500), and biotin-conjugated goat anti-mouse IgG were added and incubated overnight at 4°C. Then, a horseradish peroxidase-labeled streptavidin working solution was added [28]. DAB was adopted for coloration in the dark. The sections were dehydrated with gradient ethanol, transparentized with xylene, and mounted with neutral balsam. The organizational morphology was monitored under an Olympus light microscope (200 ×).

### 2.14. Data analysis

The SPSS17.0 statistical software (SPSS Inc., Chicago, IL, USA) was adopted for analysis. Measurement data were expressed as mean ± standard deviation (x ± s). Two-group data comparison was made by *t*-test, while analysis of variance was employed to compare the differences among multiple groups. Enumeration data were expressed by the fourfold table (or percentages), and the differences between the two groups were compared by χ2. *P*< 0.05 indicated statistical significance.

## 3 Results

### 3.1 DARS-AS1 was overexpressed in HCC tissues and cells and heralded a poor prognosis

**Figure 1.**
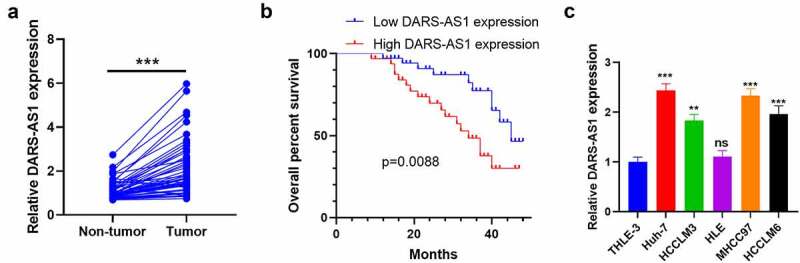
DARS-AS1 expression in HCC tissues and cells and its correlation with HCC prognosis

To understand the expression characteristics of DARS-AS1 in HCC, we monitored the DARS-AS1 expression in HCC tissues and cells by RT-qPCR and analyzed HCC patients’ prognoses accordingly. We first examined the DARS-AS1 level in 50 HCC tissues and non-tumor tissues. The results disclosed that DARS-AS1 was up-regulated in HCC tissues (vs. that in non-tumor tissues) (Figure 1a). Additionally, we analyzed the relationship between the DARS-AS1 profile and HCC prognosis. As a result, the overall survival rate of patients with high DARS-AS1 levels was reduced (Figure 1b), accompanied by larger tumor volume and more distant metastasis (Table 1). Moreover, we examined the DARS-AS1 profile in HCC cells and normal liver cell lines and discovered that the DARS-AS1 level was elevated in HCC cells (vs. normal liver cell line L-02) (Figure 1c). In conclusion, DARS-AS1 was a poor prognostic factor for HCC and was involved in HCC cell proliferation and metastasis.

**Table 1.** The correlation between the lncRNA DARS-AS1 expression level and the Pathological factors of HCC patients

Pathological factors	DARS-AS1 expression level		p value
	Low (n = 25)	High (n = 25)	
Age (year)			
<55	9	11	5637
≥55	16	14	
Gender			
Male	18	16	5442
Female	7	9	
AFP level			
≥400 μg/L	20	22	4404
<400 μg/L	5	3	
TNM stage			
I–II	19	11	0209*
III–IV	6	14	
Tumor size			
≥5 cm	8	15	047*
<5 cm	17	10	
Lymph node metastasis			
Yes	5	11	0689
No	20	14	

Note: *indicates *P*< 0.05.

A. The DARS-AS1 expression in 50 HCC tissues and non-tumor tissues was examined by RT-qPCR. *** indicated*P*< 0.001. B.Kaplan-Meier analysis was adopted to assess the correlation between the DARS-AS1 level and the overall survival rate of HCC patients. C. The DARS-AS1 profile in normal liver cell line L-02 and HCC cell lines (Huh-7 and HCC-LM3) was determined by RT-qPCR. **, *** indicated*P*< 0.01, *P*< 0.001 compared with the L-02 group. N= 3.

### 3.2 DARS-AS1 facilitated HCC cell proliferation, invasion and EMT and inhibited cell apoptosis

**Figure 2.**
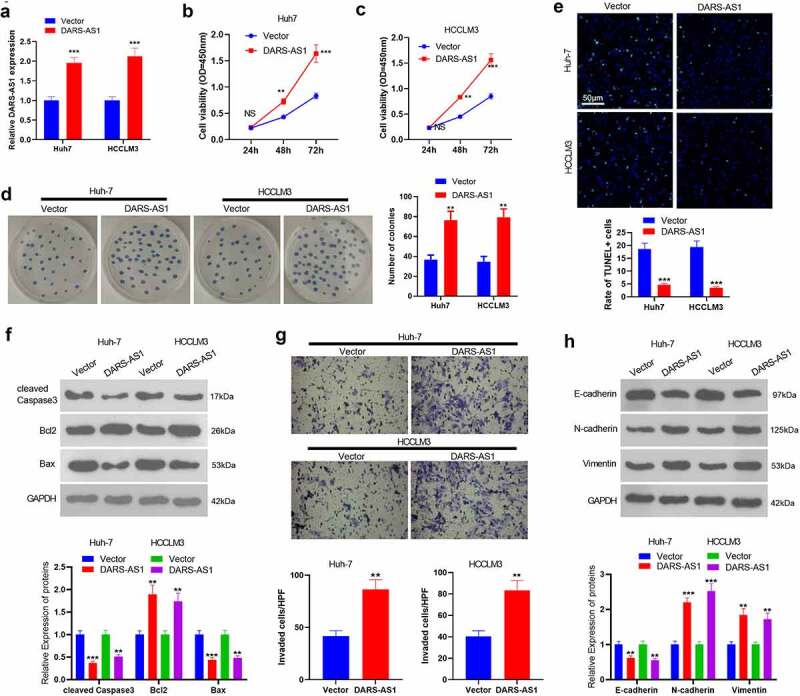
DARS-AS1 subserved HCC proliferation, invasion and EMT

According to the above data, we speculate that DARS-AS1 exerts a carcinogenic effect in HCC cell growth. Therefore, we transfected Huh-7 and HCC-LM3 cells with DARS-AS1 overexpression plasmids. RT-qPCR outcomes manifested that compared with the vector group, DARS-AS1 overexpression plasmids transfection significantly elevated DARS-AS1 levels (Figure 2a). CCK-8 experiment and colony formation assay were implemented to verify cell proliferation, and it turned out that overexpressing DARS-AS1 strengthened HCC cell proliferation and colony formation (Figure 2b-d). Cell apoptosis was tested by TUNEL assay and WB, which unveiled that the TUNEL-positive rate of DARS-AS1 overexpressed HCC cells was lower than that of the vector group (Figure 2e). Moreover, DARS-AS1 up-regulation hindered cleaved Caspase3 and Bax levels while boosted Bcl2 expression (figure 2f). Further, the Transwell assay confirmed that overexpressing DARS-AS1 heightened cell invasion (Figure 2g). EMT was an important mechanism of HCC metastasis, so we conducted WB to monitor EMT markers (including E-cadherin, N-cadherin and Vimentin) in HCC cells. It was found that overexpressing DARS-AS1 hampered the expression of epithelial cell marker E-cadherin and elevated the expression of mesenchymal markers N-cadherin and Vimentin (Figure 2h). Thus, DARS-AS1 expedited HCC evolvement by increasing HCC cell proliferation, reducing cell apoptosis, and accelerating invasion and EMT.

A. DARS-AS1 overexpression models were constructed in Huh-7 and HCC-LM3, respectively, and the DARS-AS1 expression was tested by RT-qPCR. B and C. CCK-8 experiment was implemented to verify Huh-7 and HCC-LM3 cell proliferation. D. Cell proliferation was examined by the colony formation assay. E. Cell apoptosis was monitored by the TUNEL assay. F. The expression of Caspase3, Bax and Bcl2 in HCC cells was compared by WB. G. Transwell assay was performed to test cell invasion. H. The expression of E-cadherin, N-cadherin and Vimentin in HCC cells was compared by WB. NS, **, *** represented*P*> 0.05,*P*< 0.01,*P*< 0.001, compared with the Vector group. N = 3.

### 3.3 *DARS-AS1 strengthened Huh7 cells’ growth and metastasis* in vivo

**Figure 3.**
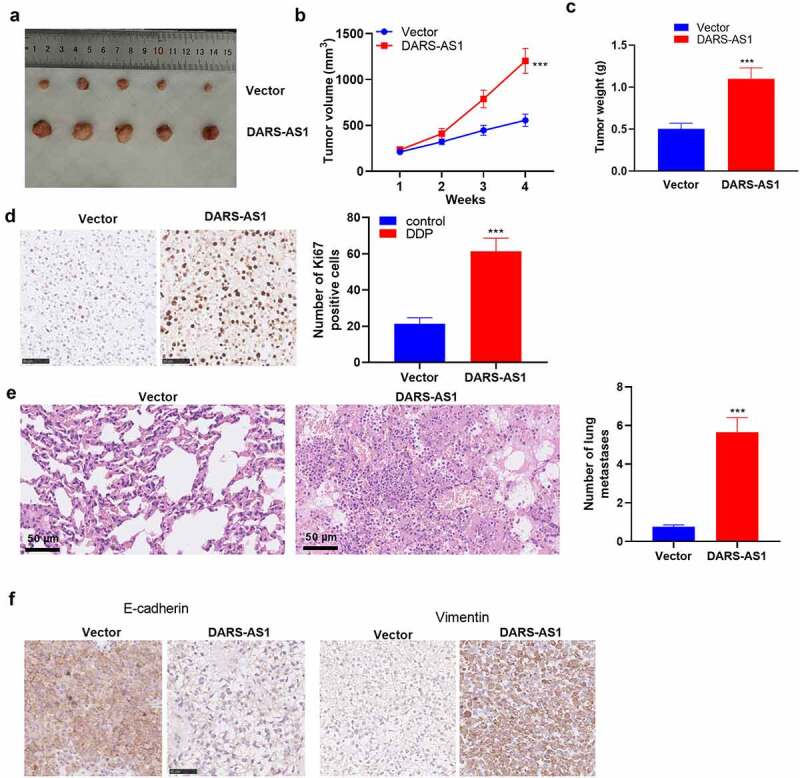
DARS-AS1 overexpression accelerated the growth and metastasis of HCC cells

To further confirm the role of DARS-AS1 in HCC cell growth, *in-vivo* experiments were conducted. A tumor-Xenograft model was set up in nude mice using Huh7 cells with DARS-AS1 overexpression. As a result, DARS-AS1-overexpressed Huh7 cells exhibited significantly enhanced growth ability (Figure 3a-c). Additionally, by contrast with the vector group, the positive rate of Ki67 in tumor tissues in the DARS-AS1 group was significantly facilitated (Figure 3d). We then utilized HE staining to evaluate the tumor cells’ lung metastasis. The outcomes uncovered that DARS-AS1 overexpression heightened lung metastasis of Huh7 cells (Figure 3e). Furthermore, IHC demonstrated that E-cadherin expression was declined and Vimentin expression was uplifted in DARS-AS1 overexpressed tissues (figure 3f). Hence, DARS-AS1 facilitated HCC cell growth and metastasis *in vivo*.

Huh7 cells were transfected with DARS-AS1 overexpression plasmids and negative control vectors. The xenograft tumor model was constructed in Huh7 cells. A. On day 28, the nude mice were killed, and the tumors were isolated. B. Tumor volume from day 7 to day 28. C. Tumor weight at day 28. N = 5. D. Ki67-positive cells were counted by IHC; N = 5. E. HE staining exhibited Huh7 metastasis to lung tissues; N = 10. F. The expression of E-cadherin and Vimentin in tumor tissues was compared by IHC. *** indicated*P*< 0.001 compared to the Vector group. N= 5.

### 3.4 DARS-AS1 heightened the CKAP2 expression

**Figure 4.**
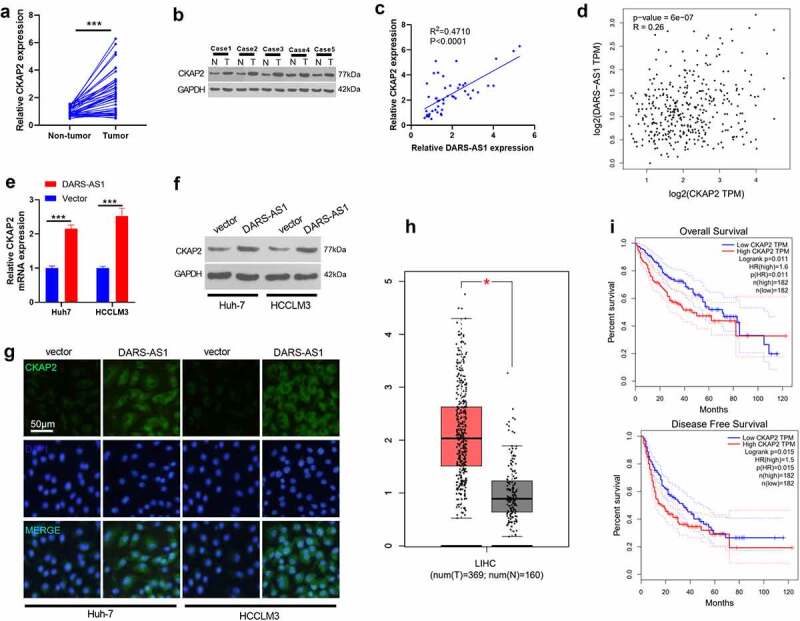
DARS-AS1 promoted the CKAP2 expression

CKAP2 has been found to exert an essential role in tumor progression [14,15,16,17]. Here, we are curious about its role in HCC and its connection with DARS-AS1. We tested the CKAP2 level in HCC tissues by RT-qPCR and WB to further probe the molecular mechanism of DARS-AS1 in regulating HCC. The results demonstrated that CKAP2 was overexpressed in HCC tissues (vs. that in non-tumor tissues) (Figure 4a, 4b). The correlation between the levels of CKAP2 and DARS-AS1 in HCC tissues was further analyzed by linear regression, and it was revealed that there was a positive correlation between the two (Figure 4c,*p*< 0.001). Besides, a significant positive correlation was found between CKAP2 and DARS-AS1 in HCC tissues through GEPIA database analysis (*P* < 0.001, Figure 4d). Meanwhile, the CKAP2 expression was determined by RT-qPCR, WB and cell immunofluorescence. It turned out that overexpressing DARS-AS1 heightened the mRNA and protein expression of CKAP2 (Figure 4e-g). Besides, the GEPIA database illustrated that CAKP2 was up-regulated in LIHC tissues (Figure 4h), and HCC patients with CKAP2 overexpression had worse overall survival and disease-free survival (Figure 4i). These findings suggested that DARS-AS1 functioned in HCC by up-regulating CKAP2.

A and B. The CKAP2 profile in HCC tissues was examined by RT-qPCR (A) and WB (B). C. Linear regression was further used to analyze the correlation between the levels of CKAP2 and DARS-AS1 in HCC tissues, which confirmed that the two were positively correlated. D. GEPIA database revealed that CKAP2 and DARS-AS1 were positively correlated in HCC tissues. E-G. The CKAP2 profile in HCC cells overexpressing DARS-AS1 was monitored by RT-qPCR, WB and cell immunofluorescence, respectively. H-I. The CKAP2 expression in LIHC tissues and non-tumor tissues was analyzed by the GEPIA database, and the relationships between the CKAP2 level and HCC patients’ overall survival and disease-free survival were analyzed. *, *** indicated P< 0.05, P< 0.001. N= 3.

### 3.5 Overexpressing CKAP2 heightened HCC cell proliferation, invasion and EMT by up-regulating FAK/ERK

**Figure 5.**
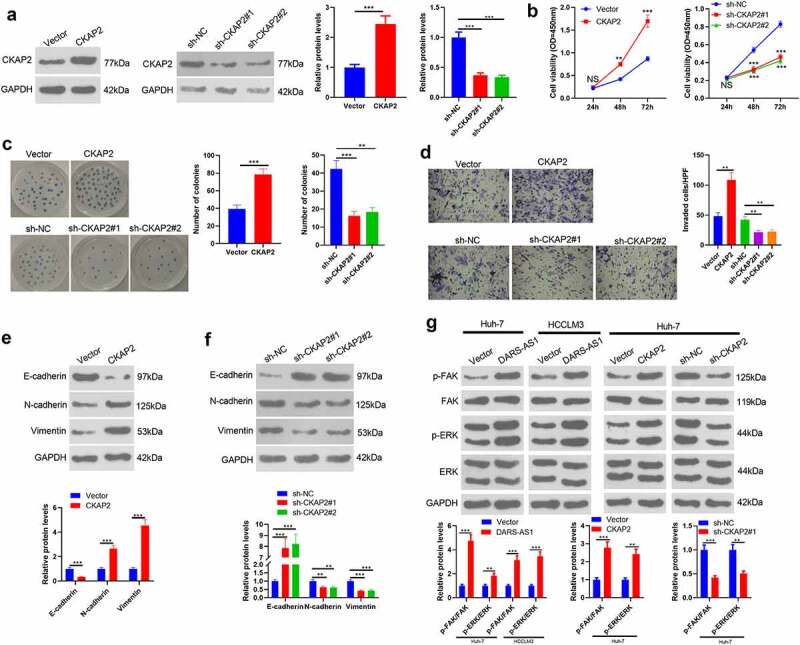
Overexpressing CKAP2 heightened the proliferation, invasion and EMT of HCC cells

Since CKAP2 was up-regulated in HCC, a conjecture that CKAP2 potentials HCC development came out. Therefore, we constructed CKAP2 overexpression and knockdown cell models to explore the influence of CKAP2 on the proliferation, invasion, and EMT of HCC cells (Figure 5a). CCK-8 experiment and colony formation assay confirmed that in comparison to the control group, up-regulating CKAP2 facilitated HCC proliferation, while knocking down CKAP2 had the opposite effect (Figure 5b-c). Additionally, the Transwell assay and WB manifested that CKAP2 overexpression amplified HCC cells’ invasion and EMT, while CKAP2 knockdown exerted the reverse effect (Figure 5d-f). WB was implemented to compare the expression of FAK and ERK in cells with different DARS-AS1 and CKAP2 levels. Interestingly, DARS-AS1 overexpression heightened FAK and ERK phosphorylation (Figure 5g). Similarly, overexpressing CKAP2 phosphorylated FAK-ERK, while knocking down CKAP2 had the opposite effect (Figure 5h). Thus, CKAP2 facilitated HCC cell proliferation, invasion and EMT through FAK-ERK, thereby accelerating HCC evolvement.

A CKAP2 overexpression and knockdown models were constructed in Huh-7 cells, respectively. WB was implemented to verify the CKAP2 expression. B. Huh-7 cell proliferation was examined by CCK-8. C. Cell proliferation was gauged by the colony formation assay. D. Transwell assay was conducted to test cell invasion. E-F. WB was applied to compare the expression of E-cadherin, N-cadherin and Vimentin in HCC cells. G The FAK/ERK profile in HCC cells transfected with DARS-AS1 overexpression plasmids was determined by WB. H WB was utilized to test the FAK/ERK expression in HCC cells transfected with CKAP2 overexpression plasmids or sh-CKAP2. NS, **, *** indicated *P*> 0.05,*P*< 0.01,*P*< 0.001. N= 3.

### 3.6 DARS-AS1 targeted miR-3200-5p, while the latter targeted CKAP2

**Figure 6.**
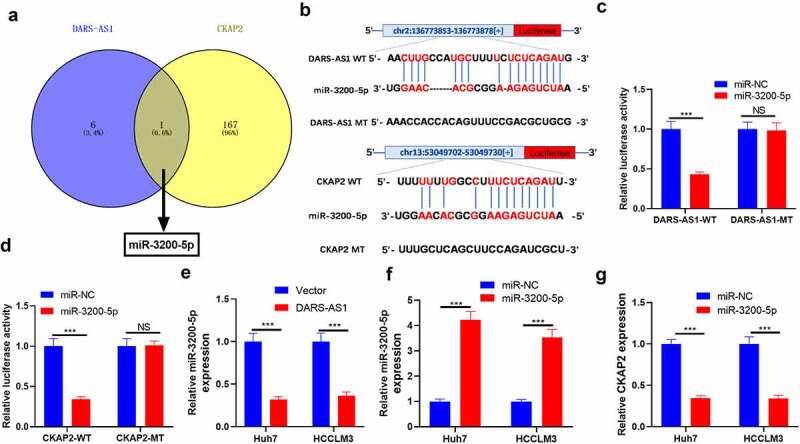
DARS-AS1 targeted miR-3200-5p, and the latter targeted CKAP2

LncRNAs regulate mRNAs by acting as a sponge of miRNAs, thereby regulating modulating progression [11,12]. Therefore, we speculate that the carcinogenic effect of DARS-AS1 in HCC may be achieved by regulating miRNAs. Therefore, We analyzed potential miRNAs binding to DARS-AS1 and CKAP2 using the Starbase database to further explore the mechanism of DARS-AS1 regulating CKAP2. Interestingly, miR-3200-5p had a common binding site with DRAS-1 and CKAP2 (Figure 6(a, b)). Therefore, we constructed dual-luciferase reporter gene vectors with miR-3200-5p binding site mutation on DARS-AS1 and CKAP2 gene fragments and transfected miR-3200-5p mimics into Huh7 cells. Next, we gauged the dual-luciferase activity of Huh7 cells. It turned out that miR-3200-5p mimics obviously impeded the luciferase activity in DARS-AS1-WT and CKAP2-WT vector-transfected cells while had no impact on DARS-AS1-MT and CKAP2-MT vector-transfected cells (Figure 6(c-d)). Then, we verified the miR-3200-5p expression in HCC cells overexpressing DARS-AS1 by RT-qPCR. We discovered that miR-3200-5p was down-regulated after DARS-AS1 overexpression (Figure 6e). In addition, CKAP2 mRNA levels were decreased in HCC cells overexpressing miR-3200-5p (Figure 6g). Overall, DARS-AS1 competitively hampered miR-3200-5p, and the latter targeted CKAP2.

A-B. The potential miRNAs binding to DARS-AS1 and CKAP2 were predicted by the Starbase database. As a result, miR-3200-5p had a common binding site with DARS-AS1 and CKAP2. C-D. The wild-type or mutated dual-luciferase reporter gene vectors of DRAS-1 and CKAP2 and miR-3200-5p mimics were transfected into Huh7 cells. E. The miR-3200-5p expression in DARS-AS1-overexpressed HCC cells was examined by RT-qPCR. F. HCC cells overexpressing miR-3200-5p were constructed by transfecting the HCC cells with miR-3200-5p mimics. G. RT-qPCR was applied to test the mRNA level of CKAP2 in the miR-3200-5p overexpression model. NS, *** indicated *P*> 0.05,*P*< 0.001. N = 3.

### 3.7 DARS-AS1 attenuated the tumor-suppressive effect mediated by miR-3200-5p overexpression

**Figure 7.**
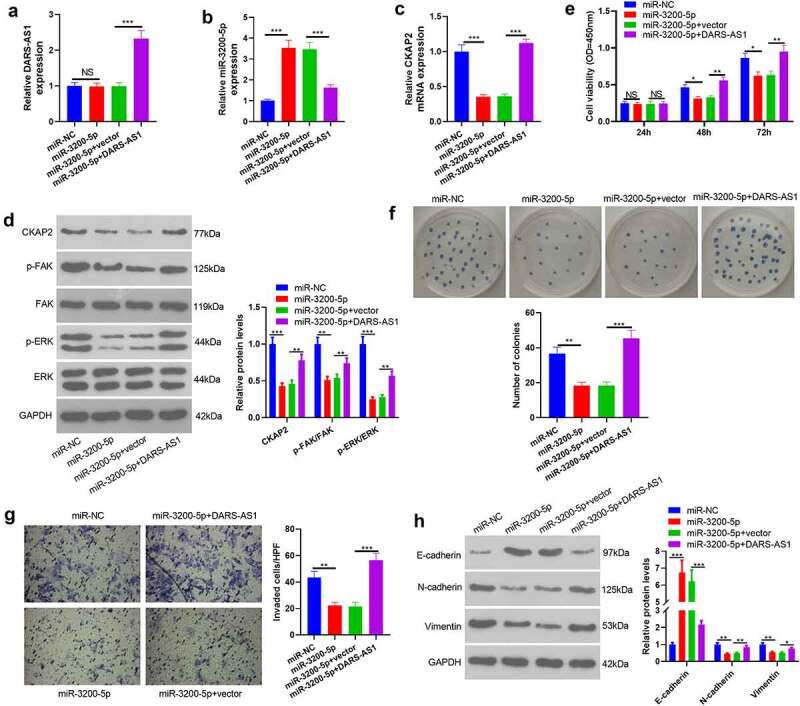
DARS-AS1 weakened the tumor-suppressive effect mediated by miR-3200-5p overexpression

The above data have confirmed that miR-3200-5p is a downstream target of DARS-AS1. However, the regulatory role of the DARS-AS1-miR-3200-5p axis in HCC remains unknown. Thus, compensation tests were performed by transfecting miR-3200-5p mimics and, or DARS-AS1 overexpression plasmids into Huh7 cells. RT-qPCR and WB outcomes disclosed that compared with the miR-3200-5p+Vector group, the levels of DARS-AS1, CKAP2 and the FAK/ERK pathway were heightened, while the miR-3200-5p expression was suppressed in the miR-3200-5p+DARS-AS1 group (Figure 7(a-d)). We further examined cell proliferation, invasion and EMT changes. The results exhibited that cell proliferation, invasion, and EMT levels were reduced in the miR-3200-5p group compared with that of the control group (miR-NC group) (Figure 7(e-h)). However, overexpressing DARS-AS1 brought about enhanced proliferation, invasion and EMT of Huh7 cells (Figure 7(e-h)). Therefore, DARS-AS1 exerted a carcinogenic effect by abating miR-3200-5p, up-regulating CKAP2, and activating the FAK/ERK pathway.

miR-3200-5p mimics or DARS-AS1 overexpression plasmids were transfected into Huh7 cells. A-D. RT-qPCR and WB were utilized to check DARS-AS1 (A), miR-3200-5p (B), CKAP2 (C) and FAK/ERK (D) levels. E. CCK-8 was performed to verify Huh-7 and HCC-LM3 cell proliferation. F. Cell proliferation was examined by the colony formation assay. G. Transwell assay was implemented to test cell invasiveness. H. The expression of E-cadherin, N-cadherin and Vimentin in HCC cells was compared by WB. NS, *, **, *** indicated *P*> 0.05,*P*< 0.05,*P*< 0.01,*P*< 0.001. N= 3.

## 4. Discussion

Multiple reports have confirmed that lncRNAs exert a dominant role in HCC. Here, we investigated the role of DARS-AS1 in regulating HCC progression. According to reports, DARS-AS1 is abnormally expressed in tumor tissues and can be used as a prognostic indicator for various tumors [9,10,11,12]. Functionally, DARS-AS1 modulates diversified cellular biological processes, including tumor cell proliferation, migration, invasion and apoptosis. Here, we found that DARS-AS1 not only served as a poor prognostic factor for HCC, but also heightened the proliferation, invasion and EMT of HCC by regulating the miR-3200-5p-CKAP2/FAK/ERK pathway. Meanwhile, DARS-AS1 was overexpressed in HCC tissues, and high levels of DARS-AS1 predicted poor prognosis and were significantly correlated with tumor volume and distant metastasis in HCC patients. *In-vitro* and *in-vivo* experiments verified that DARS-AS1 heightened HCC cell proliferation and invasion and restrained cell apoptosis. Therefore, DARS-AS1 is expected to become an important diagnostic and therapeutic target in HCC.

Similar to lncRNAs, microRNAs (miRNAs) are about 18–25 bases long, without a protein-coding function. In recent years, emerging evidence has pointed out that miRNAs regulate tumors’ malignant phenotype and then affect tumor progression [29,30,31]. In addition [32], DARS-AS1 can act as a competitive endogenous RNA to sponge miRNAs, thereby choking the effects of targeted miRNAs. DARS-AS1 facilitates the proliferation of childhood acute myeloid leukemia and thyroid cancer by sponging miR-425 and miR-129 [33]. Inspired by this, we predicted the potential miRNA targets between DARS-AS1 and CKAP2 through bioinformatics. We discovered that miR-3200-3p contained binding sites with DARS-AS1 and CKAP2. Functionally, miR-3200-5p impeded the proliferation, invasion and EMT of HCC, while DARS-AS1 mostly reversed this effect. These data further confirm the diagnostic and therapeutic value of DARS-AS1/miR-3200-5p in HCC. In fact, previous studies have also supported the inhibitory effect of miR-3200-5p on tumors, such as gastric cancer [34].

EMT is a complex cellular process characterized by its plasticity and reversibility. It plays a pivotal role in a wide array of physiological and pathological processes, such as embryonic development, organogenesis, tissue repair, fibrosis, tumor metastasis and drug resistance [35]. Effective modulation of EMT considerably weakens the distant metastasis of HCC [36]. This study stated that DARS-AS1 overexpression heightened EMT in HCC cells, which was specifically reflected in the up-regulation of the mesenchymal markers, N-cadherin and Vimentin, and the down-regulation of the epithelial marker, E-cadherin. In contrast, DARS-AS1 knockdown accelerated EMT. Therefore, DARS-AS1 regulated HCC evolvement by accelerating EMT.

CKAP, a consequential cytoskeleton protein in cells, ensures the stability of chromosomes by maintaining the integrity of microtubule nucleation sites [13]. In addition, CKAP4 is up-regulated in various tumors, including clear cell renal cell carcinoma [37] and esophageal cancer [38], which intensifies tumor cell metastasis. Meanwhile, CKAP4 also functions as a poor prognostic factor in HCC [39]. CKAP2, a major member of CKAP, serves as a marker of breast cancer proliferation and an independent predictor of its prognosis, as reported [40]. Here, we unveiled that CKAP2 was up-regulated in HCC (vs. non-tumor tissues), suggesting that CKAP2 might be involved in HCC progression. Furthermore, gain-and-loss-of-function experiments confirmed that CKAP2 heightened HCC proliferation, invasion and EMT. Collectively, CKAP2 exerted a carcinogenic function in HCC.

FAK-ERK is among the classical signaling pathways in cells. Its activation contributes to diversified diseases, including tumors. For example, Osteopontin enhances cellular autophagy by activating the FAK-ERK pathway, thus reducing early brain damage induced by subarachnoid hemorrhage [41]. Moreover, Hexokinase 2 regulates the metastasis, invasion, and cell stemness of ovarian cancer cells through the FAK/ERK1/2/MMP9/NANOG/SOX9 signal transduction pathways [42, 43]. In particular, Epimorphin enhances the invasion and metastasis of HCC in human beings by activating the FAK/ERK/MMP-9 axis [44]. Interestingly, previous findings have confirmed CKAP2’s carcinogenic effects on ovarian cancer [45] and cervical cancer [17] by activating the FAK-ERK pathway. Here, we identified that overexpressing DARS-AS1 and CKAP2 substantially up-regulated FAK and ERK phosphorylation, while knocking down DARS-AS1 or overexpressing miR-3200-5p suppressed FAK/ERK activation. Hence, we ascertained that DARS-AS1/miR-3200-5p/CKAP2 heightened HCC progression through the FAK/ERK pathway.

## 5 Conclusion

The present study discussed the influence of DARS-AS1, a novel lncRNA, on regulating HCC progression. Our research confirmed that DARS-AS1 elevated HCC proliferation, invasion, and EMT by regulating the miR-3200-5p/CKAP2 axis. Overall, this study made an in-depth exploration into the molecular mechanism of HCC in its development and laid a novel theoretical foundation for the early diagnosis and therapy of HCC.

## Data Availability

The data sets used and analyzed during the current study are available from the corresponding author on reasonable request.
